# MicroRNA-141 Represses HBV Replication by Targeting PPARA

**DOI:** 10.1371/journal.pone.0034165

**Published:** 2012-03-30

**Authors:** Wei Hu, Xuejun Wang, Xiaoran Ding, Ying Li, Xiujuan Zhang, Peiwen Xie, Jing Yang, Shengqi Wang

**Affiliations:** Beijing Institute of Radiation Medicine, Beijing, People's Republic of China; University of Kansas Medical Center, United States of America

## Abstract

MicroRNAs (miRNAs) are small non-coding RNAs that regulate gene expression primarily at the post-transcriptional level and play critical roles in a variety of physiological and pathological processes. In this report, miR-141 was identified to repress HBV expression by screening a small miRNA expressing library and synthetic miR-141 mimics could also significantly suppress HBV expression and replication in HepG2 cells. Bioinformatic analysis and experiment assays indicate that peroxisome proliferator-activated receptor alpha (PPARA) was the target of hsa-miR-141 during this process. Furthermore, knockdown of PPARA by small interfering RNA (siRNA) inhibited HBV replication similar to levels observed for miR-141. Promoter functional analysis indicated that repression of HBV replication by miR-141 mimics or siRNA was mediated by interfering with the HBV promoter functions, consistent with previous studies demonstrating that PPARA regulated HBV gene expression through interactions with HBV promoter regulatory elements. Our results suggest that miR-141 suppressed HBV replication by reducing HBV promoter activities by down-regulating PPARA. This study provides new insights into the molecular mechanisms associated with HBV-host interactions. Furthermore, this information may facilitate the development of novel anti-HBV therapeutic strategies.

## Introduction

Structurally, microRNAs (miRNAs) are small noncoding RNAs with 18–25 nucleotides in length which are processed from short stem-loop precursors encoded by plant, animal and viral gemomes. The growing miRNA database (http://www.mirbase.org) currently contains ∼2100 human miRNAs [1]. Bioinformatic analyses have suggested that miRNAs could regulate a number of genes and each mRNA could also be regulated by several miRNAs [2]. It is estimated that more than 30% of human genes are regulated by miRNAs [3]. miRNAs have been shown to play significant roles in a variety of physiological processes including organ development, cell differentiation, apoptosis and metabolism by either mediating translational arrest or degrading target transcripts [4]–[6].

Recent data has indicated that host miRNAs could be involved in host–virus interactions, and therefore could have a significant impact on the virus life-cycle [7]. Lecellier *et al.* [8] demonstrated for the first time that miR-32 possessed antiviral properties. Specifically, miR-32 was shown to inhibit primate foamy virus type 1 (PFV-1) mRNA translation and also restricted virus accumulation in cultured cells. In addition, miR-24 and miR-93 were found to target vesicular stomatitis virus (VSV) and protect mice against VSV infection [9]. In contrast, Jopling *et al.* reported that miR-122 was necessary for hepatitis C virus (HCV) replication by binding of miRNAs to the 5′ end of the viral genome [10]. HBV is a strict intracellular pathogen that infects primary hepatocytes and is the prototypic *Hepadnaviridae* family virus. HBV must utilize host cellular components and machinery as a means of completing replication and mediating pathogenesis. Therefore, we hypothesized whether hsa-miRNAs and HBV interactions could affect HBV replication.

This study screened a hsa-miRNA expression library and identified several miRNAs with the potential of interfering with HBV replication. Among the candidate miRNAs, miR-141 was further analysized by considering its good inhibition rate of HBV replication. Results presented in this report demonstrated that miRNA-141 inhibited HBV replication by reducing the transcriptional ability of HBV promoters by targeting the transcription factor peroxisome proliferator-activated receptor alpha (PPARA).

## Results

### Primary screening of HBV-replication related miRNAs

**Figure 1:**
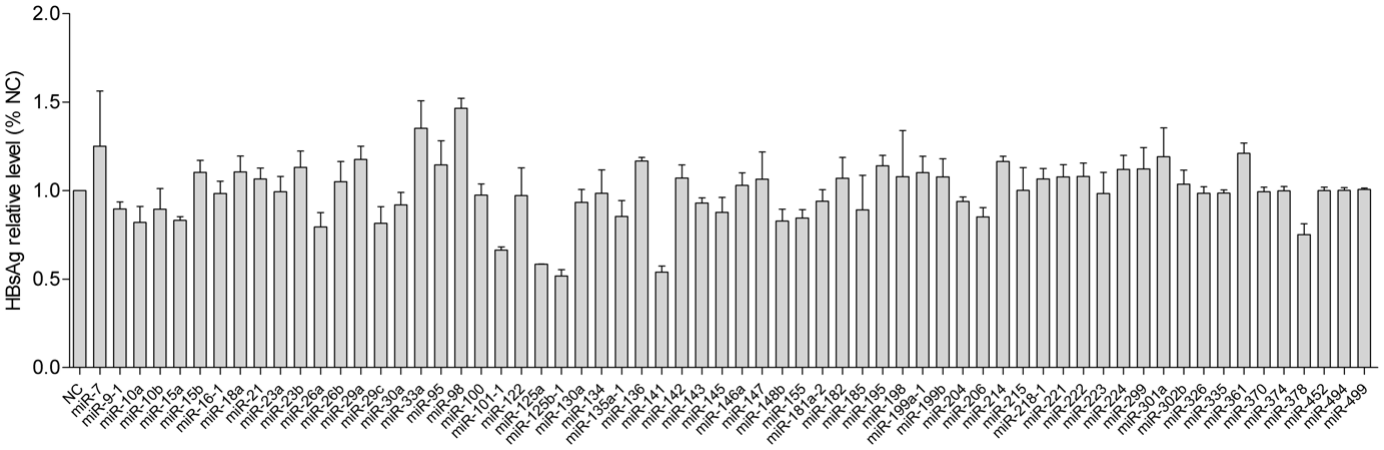
Screening for miRNAs involved in HBV expression. The negative control pcDNA3.0 plasmid and 64 miRNA expressing plasmids were co-transfected with the pHBV1.3 vector, respectively. The level of HBsAg in cell culture supernatants was detected by ELISA 48 h post transfection. The value in the negative control group was set at 1.0.

In order to identify miRNAs with the capacity of regulating HBV replication, 64 miRNAs functionally related to cell differentiation, viral infection and cancer were selected from a miRNA expression library. miRNA expression plasmids were co-transfected in triplicate into HepG2 cells with the pHBV1.3 plasmid. Cell culture supernatants were collected 48 h post transfection and screened for the presence of HBsAg by ELISA. Using this screening method, we identified miR-141, miR-125a and miR-125b could inhibit HBsAg expression in HepG2 cells, whereas miR-98 had the opposite effect (Fig. 1). Based on the screening results and for the consideration of finding effective cellular anti-HBV miRNA, we subsequently selected miR-141 for the following HBV replication inhibition research.

### miR-141 mediated inhibition of HBV expression and replication

**Figure 2:**
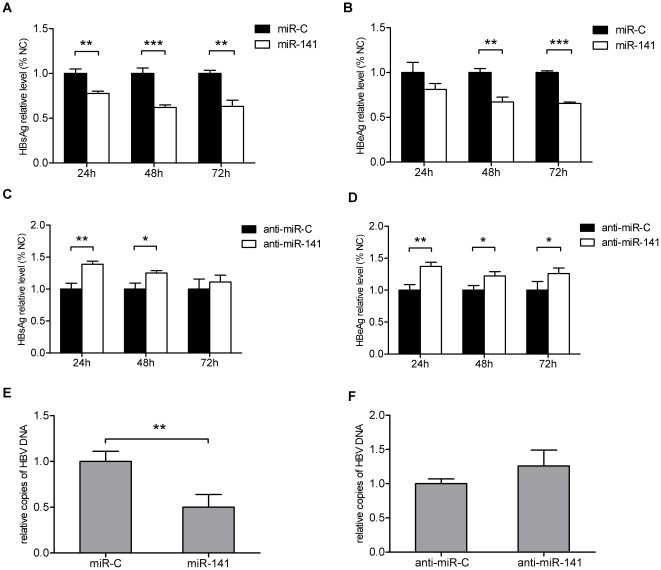
Effect of transfection with miR-141 mimics. Transfection of HepG2 cells with miR-141 mimics represses HBV expression and replication. By contrast, transfection with miR-141 inhibitor had the opposite effect. HepG2 cells were co-transfected with miR-141 mimics or miR-141 inhibitor together with the pHBV1.3 vector and analyzed for HBV protein expression. Oligonucleotides with scrambled sequences were used as negative controls (miR-C or anti-miR-C, respectively). HBsAg and HBeAg ELISA assays were used to screen culture supernatants 24, 48 and 72 h after co-transfection with (A) miR-141 mimics and the pHBV1.3 vector or (B) miR-141 inhibitor and the pHBV1.3 vector. (C) qPCR detection of HBV DNA in HepG2 cells 72 h after co-transfection with the miR-141 mimics or miR-141 inhibitor together with pHBV1.3. The histograms show the relative HBsAg, HBeAg and HBV DNA levels compared to the negative control group.

miR-141 was further examined to confirm its ability to interfere with HBV replication by co-transfecting miR-141 mimics or miR-141 inhibitor into HepG2 cells with pHBV1.3 plasmid. HBsAg/HBeAg expression levels in cell culture supernatants were analyzed by ELISA and viral DNA isolated and analyzed by quantitative PCR (qPCR). These analyses confirmed that miR-141 could repress HBV replication effectively, and that miR-141 inhibitor transfection resulted in a pronounced increase in HBsAg/HBeAg expression had no significant effect on HBV DNA replication (Fig. 2). Both of these positive and negative activities indicated that miR-141 could repress HBV replication.

**Figure 3:**
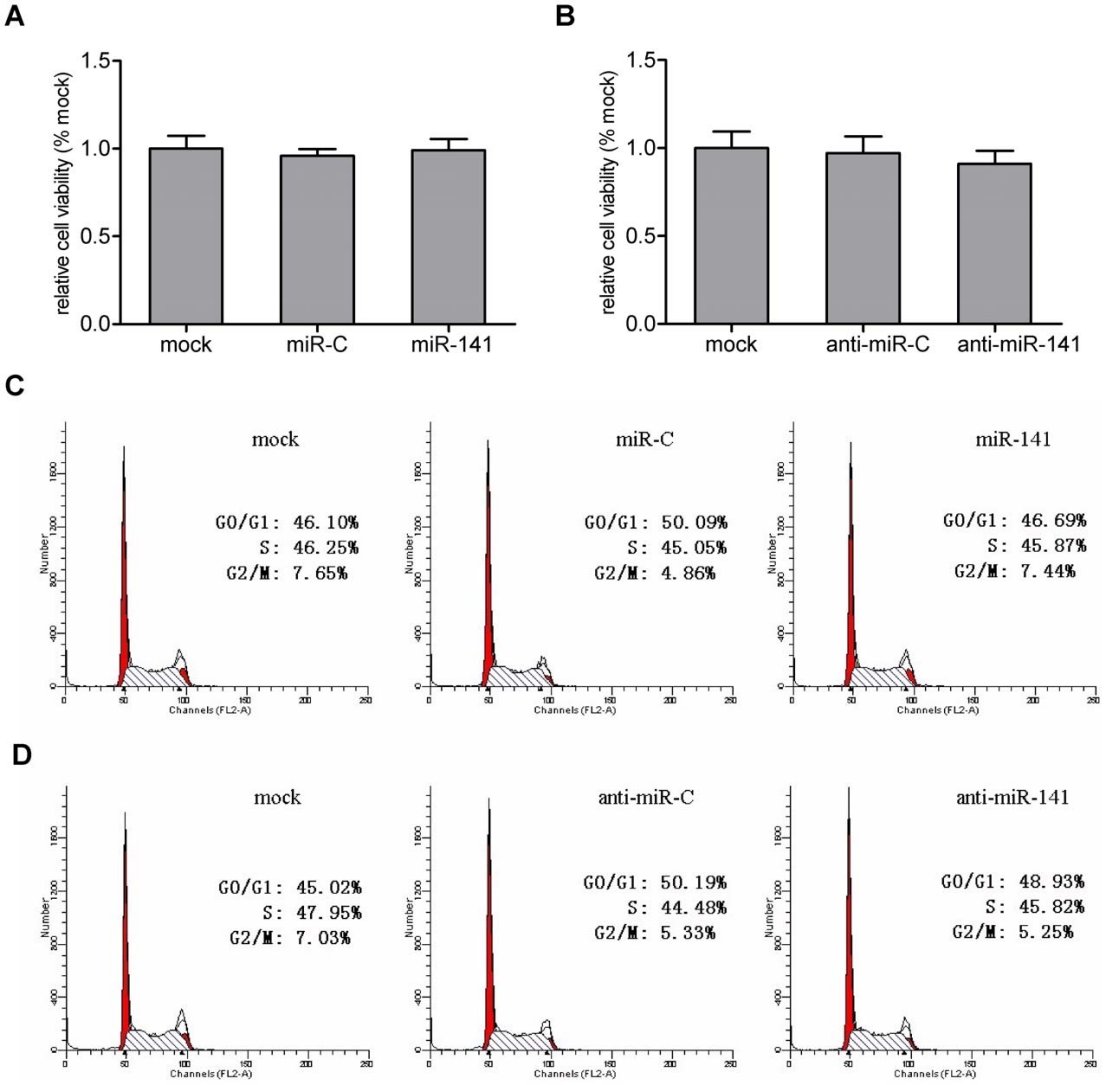
Transfection of miR-141 mimics or miR-141 inhibitor has no significant influence on HepG2 cell viability or cell cycle progression. HepG2 cells were transfected with miR-141 mimics or miR-141 inhibitor only and analyzed 72 h post transfection. Scrambled oligonucleotides were used as negative controls (miR-C or anti-miR-C, respectively). (A) HepG2 cell viability was measured using the CCK-8 assay. Data are expressed as fold change relative to mock treated cells. (B) HepG2 cell cycle analysis was performed by flow cytometry. Percent cells in each phase of the cell cycle are shown.

We next characterized the effect of miR-141 transfection related side-effects on host cells using the Cell Counting Kit-8 (CCK-8) and flow cytometry (FCM) to analyze cell viability and cell cycle progression, respectively. CCK-8 analysis showed that miR-141 mimics, as well as miR-141 inhibitor, had no significant effect on cell viability compared to negative control or mock treated cells (Fig. 3A, B). Flow cytometric analysis also showed that no significant differences in cell cycle progression could be observed between these experimental groups (Fig. 3C, D). These results validated the hypothesis that miR-141 could repress HBV replication without harming host cells.

### PPARA is a target of miR-141 in HBV-replication repression

**Figure 4:**
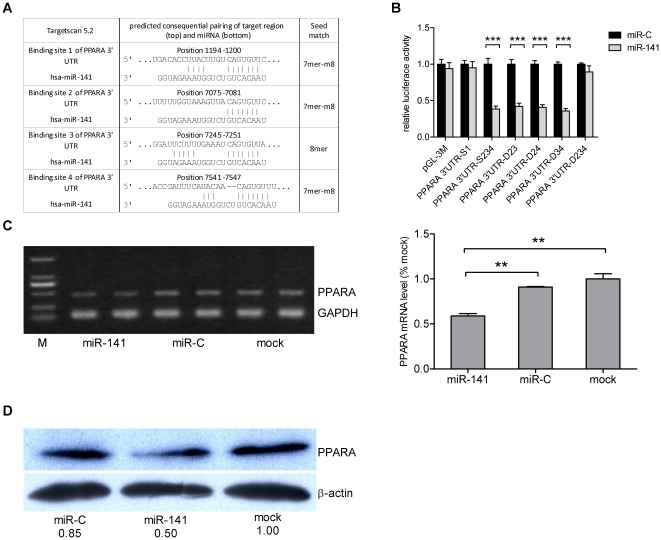
PPARA is the target of miR-141. (A) Four possible miR-141 target sites on the PPARA 3′-UTR were predicted by TargetScan software. (B) Putative miR-141 target sites 2, 3, and 4 in the PPARA 3′-UTR were functional in a dual-luciferase reporter assay. miR-141 mimics or a miRNA control were co-transfected with the luciferase reporter vector containing wild-type binding site 1 or binding sites 2, 3, and 4 of the PPARA 3′-UTR or different PPARA 3′-UTR deletion constructs containing only one binding site, respectively. Luciferase activity was determined 48 h after transfection. The histogram shows the normalized Firefly/Renilla luciferase activities as relative values compared to the miR-C negative control. Regulation mediated by miR-141 mimics on PPARA mRNA and protein expression were analyzed, respectively, by (C) semi-quantitative RT-PCR or (D) Western blot. GAPDH and β-actin were used as internal controls, respectively. The ratio of the PPARA to GAPDH or β-actin band intensities are shown. The value of mock transfected cells was set at 1.0. (Lane M, molecular weight standards).

The results from the target gene prediction program TargetScan (www.targetscan.org) [3], [11], [12] revealed that PPARA (a key liver-enriched transcription factor required for HBV pregenomic RNA synthesis and viral replication) might be a candidate miR-141 gene target. Moreover, no putative target site for hsa-miR-141 was found in the HBV genome by the computational analysis. As shown in Fig. 4A, sequence analysis revealed that there were 4 candidate miR-141 binding sites in the PPARA mRNA 3′-untranslated region (UTRs) suggesting that PPARA was probably a miR-141 target gene.

The location of the first miR-141 binding site (binding site 1) is relatively distant from binding sites 2, 3 and 4 in the PPARA 3′-UTR. Binding site 1 and binding sites 2, 3 and 4 were cloned into the pGL3M vector separately as a means of identifying the miR-141 binding site(s). The pGL3M-UTR vectors were then co-transfected with miR-141 mimics (or the negative control) into HEK293T cells. Luciferase assay results indicated that miR-141 significantly reduced the luciferase activity of the reporter plasmid containing binding sites 2, 3, and 4 but had no effect on the plasmid containing binding site 1 only (Fig. 4B). These results suggested that miR-141 targeted binding site 2, 3 or 4 of the PPARA 3′-UTR. Subsequently, different deletants of PPARA 3′-UTR containing only one of binding site 2, 3 or 4 were cloned into the pGL3M vector. Luciferase results indicated that miR-141 significantly reduced the luciferase activity of the 3 plasmids when 2 binding sites were deleted but had no effect on plasmid missing all the 3 binding sites (Fig. 4B). These results demonstrated that miR-141 regulated PPARA expression by targeting PPARA 3′-UTR binding sites 2, 3 and 4 (with no significant differences in their binding ability to miR-141).

To analyze interactions between miR-141 and PPARA in hepatocytes, miR-141 mimics was transfected into HepG2 cells and PPARA expression levels were detected by semi-quantitative RT-PCR and Western blot analyses. These results showed that miR-141 mimics markedly reduced both PPARA mRNA and protein levels compared to levels observed in the negative control miR-C transfected or mock treated cells (Fig. 4C, D). These results above indicated that miR-141 could regulate the expression of PPARA.

### Verification of PPARA function in HBV replication

**Figure 5:**
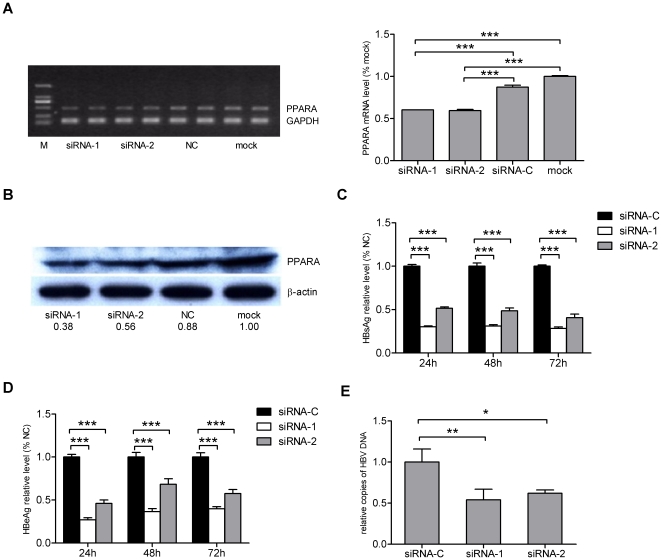
Silencing of PPARA by siRNA represses HBV replication in HepG2 cells. The levels of PPARA mRNA and protein in HepG2 cells 48 h post transfection with PPARA specific siRNA were analyzed, respectively, by (A) semi-quantitative RT-PCR or (B) Western blot. GAPDH and β-actin were used as internal controls, respectively. The ratio of the band intensities were determined as described above (Lane M, molecular weight standards). The levels of (C) HBsAg and (D) HBeAg were determined 24, 48 and 72 h post transfection. (E) HBV DNA concentrations were determined 72 h post transfection. Relative levels were normalized as a percentage of the negative control siRNA-C.

Although the importance of PPARA-RXR heterodimers in HBV replication has been reported previously [13]–[15], we confirmed these observations using our cell transfection model by silencing PPARA. HepG2 cells were transfected with PPARA-specific siRNAs only or with the pHBV1.3 plasmid. PPARA expression levels were determined by semi-quantitative RT-PCR and Western blot analyses and the HBsAg/HBeAg levels in cell culture supernatants, as well as viral DNA loads within cells, were determined as described above. Results indicated that transfection of PPARA-specific siRNAs led to a significant decrease in PPARA levels (Fig. 5A, B) and that reductions in PPARA levels resulted in a significant inhibition of HBV replication in HepG2 cells (Fig. 5C–E).

### miR-141 inhibits HBV replication by repressing HBV promoters

**Figure 6:**
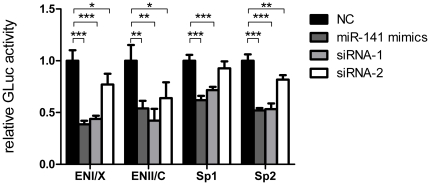
The influence of miR-141 on the activity of HBV ENI/Xp, ENII/Cp, Sp1 and Sp2 promoters. Gaussia and Renilla luciferase activities were determined 48 h post transfection. The data normalized to Renilla luciferase activity were expressed as the percentage of the negative control. miR-141 mimics and siRNA used the same oligonucleotide for the negative control.

Previous studies indicated that nuclear receptor PPARA activated HBV gene expression by interacting with HBV promoters [16]–[18]. We further verified the relationship between miR-141 and PPARA by carrying out promoter functional assays. The 4 HBV promoter fragments (ENI/Xp, ENII/Cp, Sp1, and Sp2) were cloned into the pGLuc-basic luciferase reporter vector, respectively, and then co-transfected into HepG2 cells in the presence of miR-141 mimics or PPARA-specific siRNAs. Luminescence results demonstrated that miR-141 and siRNAs significantly reduced Gaussia luciferase expression compared to the negative control in the 4 promoter functional assays (Fig. 6).

## Discussion

MicroRNAs are important small non-coding RNAs that primarily mediate post-transcriptional gene regulation. Increasing evidence suggests that there are complex interactions between cellular miRNAs and viral genes, suggesting that important regulatory networks exist between the virus, host miRNAs, and their respective target genes [8]–[10], [19]–[21]. In this report, we studied the effects of cellular miRNAs on HBV replication and found that miR-141 possessed antiviral properties.

Recent data suggested that the cellular miRNAs could regulate HBV propagation directly or indirectly by targeting cellular factors. Potenza *et al.* demonstrated that hsa-miR-125a-5p interacted with HBV sequences and repressed HBsAg expression [22]. Our primary screening of HBV replication-related miRNAs showed similar results, indicating that a pri-miR-125a expression vector could repress HBsAg synthesis in HepG2 cells. Although miR-125a-5p was shown to repress HBsAg expression by targeting HBV sequences directly, interactions between host cell proteins and miR-125a-5p could not be excluded. On the other hand, Lu *et al.* recently demonstrated that miR-1 enhanced HBV core promoter activity by increasing the expression levels of the farnesoid X receptor alpha (FXRA), an essential HBV cellular transcription factor [23]. In the present study, exogenous expression of miR-141 could significantly inhibit HBV expression and DNA replication, whereas miR-141 inhibitor transfection had the opposite effects on HBsAg/HBeAg expression. Though the regulation of HBV replication by miR-141 inhibitor is less effective than miR-141 mimics, it is possible due to the relative low expression level of miR-141 in HepG2 cells (Information S1). These data suggest that miR-141 could suppress HBV transcription and replication.

HBV contains a 3.2 kb partially double-stranded DNA genome with four promoters (the core, pre-S1, pre-S2/S, and X promoters) and two enhancer regions (ENI and ENII) involved in viral transcription regulation [24], [25], and transcriptional regulation plays a central role in control of HBV replication [26]. HBV promoter and enhancer activities are regulated by a number of transcription factors [27]. For example, the core promoter, pre-S1 promoter, X promoter, ENI and ENII all contain a PPARA binding site and these regions were found to be transactivated in the presence of RXRA and PPARA [17]. Tang and McLachlan also demonstrated that nuclear hormone receptors (NRs), including HNF4, RXRA and PPARA, were essential liver-enriched transcription factors associated with HBV pregenomic RNA synthesis and viral replication in nonhepatic cell lines [14]. Since pregenomic RNA encodes both polymerase and core protein (and serves as the template for viral DNA synthesis), it is likely that PPARA plays a critical role in HBV biogenesis.

The peroxisome proliferator-activated receptors (PPARs) that consist of PPARα, PPARβ, and PPARγ are ligand-activated transcription factors that belong to the nuclear hormone receptor superfamily [28], [29]. PPARs serve as lipid metabolism regulators and influence cellular proliferation, differentiation and apoptosis [30], [31]. The luciferase reporter assay showed that miR-141 suppressed reporter activity significantly via 3 binding sites in the PPARA 3′-UTR. PPARA mRNA and protein levels were both reduced by miR-141 mimics transfection. This suggested that miR-141 would be an effective regulator of HBV replication by interfering with PPARA expression. Furthermore, our results showed that PPARA knockdown by miR-141 mimics or siRNA exerted a negative effect on HBV replication and reduced the HBV promoter transcription activities. This was consistent with previous findings regarding PPARA functional activity, suggesting that inhibition of HBV replication by miRNA-141 was mainly mediated via PPARA repression. Therefore, overexpression of miR-141 may be an effective strategy for diminishing HBV replication.

In conclusion, our data suggested that miR-141 down regulated HBV expression and replication by targeting cellular nuclear factor PPARA, and that PPARA could be a promising host-oriented drug target for the development of novel HBV therapy. Considering the importance and complexity of miRNAs in virus-host regulatory networks, further systematic studies will be necessary to fully unravel the role of miRNAs in viral replication. This would not only increase our knowledge regarding HBV pathogenesis mechanisms but also help in the development of novel antiviral therapeutic approaches.

## Materials and Methods

### Reagents

hsa-miR-141 2′-O-methyl (2′-OMe) mimic oligonucleotides, hsa-miR-141 inhibitor, PPARA-specific siRNAs and unrelated sequence negative controls were purchased from Genepharma (Shanghai, China). Sense and antisense PPARA siRNAs sequences were: siRNA-1, 5′-CCAAUGGCAUCCAGAACAA dTdT and 5′-UUGUUCUGGAUGCCAUUGG dTdT; siRNA-2, 5′-GCAAUGGACCAUGUAACAA dTdT and 5′-UUGUUACAUGGUCCAUUGC dTdT, respectively. DNA oligonucleotides were synthesized by Sangon Biotech (Shanghai, China). An expression library of 64 miRNAs was obtain from Xiaofei Zheng (Beijing Institute of Radiation Medicine) as described previously [32]. The HBV replication-competent vector pHBV1.3 containing 1.3 copies of the HBV genome (ayw subtype, GenBank accession number: V01460) was provided by Dr. Hua Tang (Chongqing Medical University, Chongqing, China).

### Cell culture and transfection

HepG2 and HEK293T cells (obtained from the American Type Culture Collection, Manassas, VA) were cultured in Dulbecco's Modified Eagle Medium (DMEM, GIBCO) containing 10% fetal bovine serum (FBS, Hyclone), 100 U/ml penicillin and 100 µg/ml streptomycin and maintained at 37°C in a humidified 5% CO_2_ atmosphere. Plasmids, miRNAs and siRNAs were co-transfected into cells at the indicated concentrations using Lipofectamine 2000 (Invitrogen, Carlsbad, CA) following the manufacturer's protocol 24 h after plating.

### HBV replication analysis

Cell culture media was changed and collected at 24, 48 and 72 h post transfection and centrifugated at 500×g for 5 min to remove debris before analysis. Supernatant HBsAg and HBeAg levels were determined using ELISA kits (Kehua Biotech, Shanghai, China). HBV DNA from intracellular core particles was extracted at 72 h post transfection as described previously [33]. Cells were lysed with 0.2 ml 0.5% Nonidet P-40 in 50 mM Tris-HCl and 1 mM EDTA (pH 8.0) for 10 min. Lysates were centrifuged at 1,000×g for 1 min to remove nuclei and the supernatants centrifuged for an additional 5 min at 14,000×g to clear cellular debris. Supernatants were then readjusted to 5 mM CaCl_2_ and digested with 800 units/ml micrococcal nuclease (New England Biolabs, Ipswich, MA) for 2 h at 37°C to eliminate residual plasmid DNA and unencapsidated HBV RNA. After nuclease inactivation using EDTA (10 mM), viral DNA was extracted using the Column Viral DNAout kit (TIANDZ, China) following the manufacturer's protocol and quantified by real-time PCR as described previously [34].

### Western blot analysis

Total HepG2 cellular proteins were prepared in RIPA buffer (50 mM Tris-HCl, pH 7.4, 150 mM NaCl, 0.1% SDS, 1% NP-40) containing a protease inhibitor cocktail (Roche Molecular Biochemicals, Mannheim, Germany). Polyacrylamide gel electrophoresis (PAGE) and protein transfer to Hybond polyvinylidene difluoride membranes (Amersham, Arlington Heights, IL, USA) were carried out following standard protocols. Monoclonal antibody against PPARA (sc-130640, Santa Cruz, USA) and β-actin (sc-1616-R, Santa Cruz) were used for immunodetection according to the manufacturer's instructions. Protein bands were visualized by autoradiogram using ECL Plus Western blot detection reagents (GE Healthcare Life Sciences) and quantified using Gel Pro Analyzer software v4.0 (Media Cybernetics, Bethesda, MD).

### RNA extraction and semi-quantitative RT-PCR

Total RNA was extracted using TRIzol Reagent (Invitrogen) as recommended by the manufacturer. Semi-quantitative RT-PCR using a 2-step method was used to determine PPARA mRNA expression levels. Reverse transcription was performed following the SuperScript™ III Reverse Transcriptase (Invitrogen) protocol. PCR was performed for PPARA amplification using the following conditions: 94°C for 4 min followed by 29 cycles at 94°C for 20 s, 55°C for 20 s, and 72°C for 40 s with a final extension at 72°C for 5 min and for GAPDH amplification using the following conditions: 94°C for 4 min followed by 27 cycles at 94°C for 20 s, 55°C for 20 s, and 72°C for 40 s with the final extension at 72°C for 5 min. DNA products were analyzed by 1.0% agarose gel electrophoresis and visualized following ethidium bromide staining under UV light and band intensities measured by scanning with Gel Doc 1000 (Bio-Rad, Hercules, CA). The products were quantified by densitometry, and GAPDH mRNA levels used for normalization. The primers used for PPARA and GAPDH amplification were: PPARA-F, 5′-CCTCTCAGGAAAGGCCAGTA-3′, PPARA-R, 5′-TCCACAGCAAATGATAGCAG-3′, GAPDH-F, 5′-GTCAAGCTCATTTCCTGGTATG-3′ and GAPDH-R, 5′-CTTCCTCTTGTGCTCTTGCTG-3′.

### Luciferase reporter assays

During the miRNA-target validation test, the 3′-UTRs containing the miR-141 predicted target sites were amplified by PCR from HepG2 cell genomic DNA and cloned into a modified pGL3-control plasmid (pGL3M) as described previously [32]. HEK293T cells were co-transfected with 200 ng of pGL3M-UTR constructs and 10 pmol miRNA mimics or a negative control per well in 24-well plates using the Lipofectamine™ 2000 transfection reagent. pRL-CMV (Promega, Madison, WI) was co-transfected as a normalization control. Luciferase activity assays were performed 48 h post transfection using the Dual-Luciferase Reporter Assay System (Promega).

During promoter functional analysis using dual-luciferase promoter assays, PCR fragments containing HBV promoters (ENI/Xp, nt 957–1354; ENII/Cp, nt 1627–1878; Sp1, nt 2704–2823; Sp2, nt 2978–3207) [35] were cloned into the pGLuc-Basic vector (New England Biolabs) upstream of the secretory Gaussia princeps luciferase, respectively. HepG2 cells were co-transfected with 500 ng of pGLuc-promoter constructs and 20 pmol miRNA mimics or PPARA specific siRNAs per well in 24-well plates using Lipofectamine™ 2000 transfection reagent with pRL-CMV as a normalization control. Dual-luciferase assays were carried out 48 h post transfection according to the manufacturer's protocol (New England Biolabs).

### Cell viability and cell cycle analysis

HepG2 cells were transfected with miR-141 mimics or miR-141 inhibitor only. Cell viability was determined using the Cell Counting Kit-8 kit (Dojindo, Kumamoto, Japan) 72 h after transfection. For cell cycle analysis, cells were harvested, combined, washed once in phosphate-buffered saline (PBS), and then fixed in 70% ethanol overnight 72 h after transfection. Staining for DNA content was performed with 50 µg/ml propidium iodide and 1 mg/ml RNase A at 37°C for 30 min. Stained cells were analyzed for cell cycle distribution on a FACScalibur flow cytometer (Becton Dickinson, USA).

### Statistical analysis

The data presented are expressed as mean ± standard deviation (SD) and statistical significance was determined by the Student's *t* test or one-way ANOVA. *P*-values are indicated by asterisks (***P<0.001, **P<0.01, *P<0.05).

## Supporting Information

Information S1 **The expression level of miR-141 was relative low in HepG2 cells.** Total RNA was extracted from the cultured cells using Trizol Reagent (Invitrogen) and the expression levels of small RNAs were confirmed by quantitative RT-PCR using miRNA Real-Time PCR Assay kit (CW Biotech, China) according to the manufacturer's protocol. The expression level of miR-141 was relative low compared with miR-24 which is expressed constitutively in HepG2 cells. The forward primer of RNAs amplification was RNA specific as shown in the table, and the reverse primer was a universal primer containing in the kit. The U6 snRNA expression level was used for normalization.
(XLS)
