# Correction: MicroRNA-141 Represses HBV Replication by Targeting PPARA

**DOI:** 10.1371/annotation/cbbe9454-0b72-44b3-a972-10dcaf22db68

**Published:** 2012-07-10

**Authors:** Wei Hu, Xuejun Wang, Xiaoran Ding, Ying Li, Xiujuan Zhang, Peiwen Xie, Jing Yang, Shengqi Wang

There is an error in Figure 2. The correct Figure 2 image can be seen here: 

**Figure:**
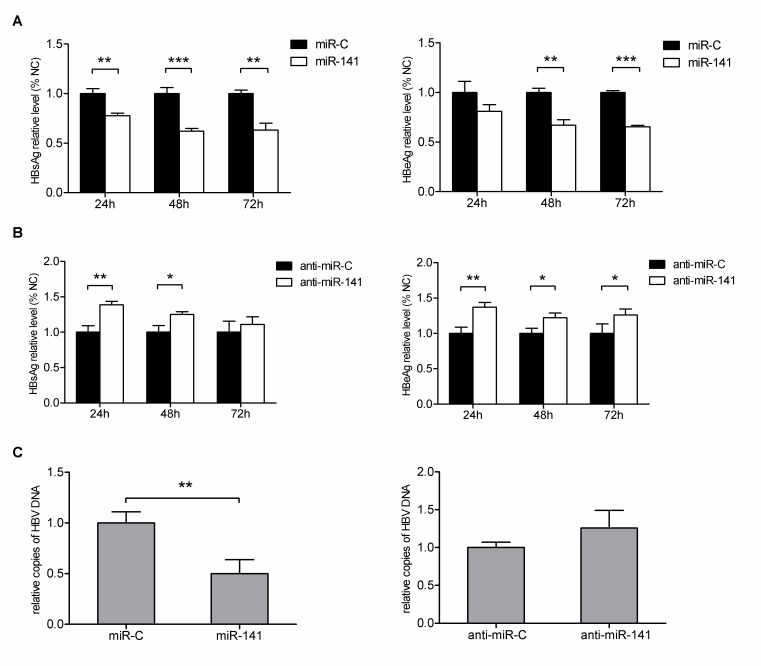


[^] 

